# TBR-760, a Dopamine-Somatostatin Compound, Arrests Growth of Aggressive Nonfunctioning Pituitary Adenomas in Mice

**DOI:** 10.1210/endocr/bqaa101

**Published:** 2020-06-27

**Authors:** Heather A Halem, Ute Hochgeschwender, Jeong Keun Rih, Richard Nelson, G Allan Johnson, Arunthi Thiagalingam, Michael D Culler

**Affiliations:** 1 Tiburio Therapeutics, Cambridge, Massachusetts; 2 College of Medicine, Central Michigan University, Mt. Pleasant, Michigan; 3 Scientific Intelligence Analytics & Modelling, Biometry R&D, Ipsen Bioscience, Cambridge, Massachusetts; 4 formerly of Ipsen Bioscience, Cambridge, Massachusetts; 5 Duke University Medical Center, Durham, North Carolina; 6 Translational Sciences, Oncology and Biomarkers, Ipsen Bioscience, Cambridge, Massachusetts

**Keywords:** TBR-760, nonfunctioning pituitary adenoma, dopastatin, dopamine, somatostatin, BIM-23A760

## Abstract

TBR-760 (formerly BIM-23A760) is a chimeric dopamine (DA)-somatostatin (SST) compound with potent agonist activity at both DA type 2 (D2R) and SST type 2 (SSTR2) receptors. Studies have shown that chimeric DA-SST compounds are more efficacious than individual DA and/or SST analogues, either alone or combined, in inhibiting secretion from primary cultures of human somatotroph and lactotroph tumor cells. Nonfunctioning pituitary adenomas (NFPAs) express both D2R and SSTR2 and, consequently, may respond to TBR-760. We used a mouse model with the pro-opiomelanocortin (POMC) gene knocked out that spontaneously develops aggressive NFPAs. Genomic microarray and DA and SST receptor messenger RNA expression analysis indicate that POMC KO mouse tumors and human NFPAs have similar expression profiles, despite arising from different cell lineages, establishing POMC KO mice as a model for study of NFPAs. Treatment with TBR-760 for 8 weeks resulted in nearly complete inhibition of established tumor growth, whereas tumors from vehicle-treated mice increased in size by 890 ± 0.7%. Comparing TBR-760 with its individual DA and SST components, TBR-760 arrested tumor growth. Treatment with equimolar or 10×-higher doses of the individual SST or DA agonists, either alone or in combination, had no significant effect. One exception was the lower dose of DA agonist that induced modest suppression of tumor growth. Only the chimeric compound TBR-760 arrested tumor growth in this mouse model of NFPA. Further, significant tumor shrinkage was observed in 20% of the mice treated with TBR-760. These results support the development of TBR-760 as a therapy for patients with NFPA.

Nonfunctioning pituitary adenomas (NFPAs) are tumors that arise in the pituitary gland, typically from gonadotrophs (1). Clinically and biochemically, these tumors are not associated with a hormone hypersecretion syndrome and thus, often present as macroadenomas that cause clinical signs and symptoms due to mass effect given the location of the pituitary near critical anatomical structures. These signs and symptoms include visual defects, headache, pituitary hormone deficiencies and occasionally, cranial nerve deficits (2). NFPA prevalence is currently estimated to be 7 to 41.3 cases per 100 000 people, with a standardized incidence rate of 0.65 to 2.34 per 100 000 (3-5). First-line treatment for patients with NFPAs is neurosurgery, typically transsphenoidal surgery (TSS); however, owing to local invasiveness or risk to local anatomical structures, complete resection is often not achievable (6-8). Regrowth of remnants or recurrence occurs in about 50% of patients; therefore, TSS is not curative 6-8). The high rate of recurrence post-TSS leads to the need for continued and potentially lifelong clinical and radiologic monitoring of patients (7). At present, treatment options for remnant growth or tumor recurrence are limited to repeated neurosurgical intervention, radiotherapy, or both (2, 8, 9). Regrowth can occur in all patients, even those without measurable postsurgical remnant (10-12). Radiation therapy may be required as adjuvant therapy to neurosurgery; however, radiation therapy is associated with permanent hypopituitarism of one or all pituitary hormone axes and also requires long-term continued surveillance of patients. Damage to the optic chiasm, stroke and other neurologic complications, as well as secondary brain tumors, are also potential complications of radiation therapy (9, 13, 14). Currently, there are no approved medical therapies for NFPAs.

NFPAs have been shown to express high levels of dopamine (DA) type 2 receptor (D2R) and moderate levels of somatostatin (SST) receptors 2 and 3 (SSTR2 and SSTR3, respectively) (15-18). Consequently, both DA and SST analogues have been studied for their efficacy in the treatment of NFPAs. Several retrospective studies have been performed and have shown promising results (1, 19). In a historical cohort analysis, postoperative patients were either treated with a DA agonist (bromocriptine or cabergoline) or monitored without treatment (19). Of those patients receiving DA agonist therapy, one group of patients began treatment when a tumor remnant was detected on the first postoperative magnetic resonance imaging (MRI) (preventive), and another group began treatment when tumor remnant progression was detected (remedial) (19). It was found that tumor control was achieved in 87.3% of patients with preventive treatment, 58.4% with remedial treatment, and 46.7% in the control group without treatment (19). Another smaller study showed that combination therapy for 6 months with both an SST analogue (octreotide) and a DA analogue (cabergoline) in 10 patients with NFPAs resulted in tumor volume reduction by a mean of 30% in 6 patients, no change in 3 patients, and an increased volume in 1 patient (20). In 2019, the first prospective study was performed, which was an open-label, randomized, parallel study of patients with residual NFPAs after TSS tumor resection (21). Patients were either treated with the DA agonist cabergoline or monitored without treatment (nonintervention group). In the cabergoline treatment group, significant tumor shrinkage was observed in 28.8% vs 10.5% of patients in the nonintervention group, stabilization of tumor was observed in 66.1% vs 73.7%, and growth in 5.1% vs 15.8% of patients, respectively, after 24 months. Although both DA and SST analogues can affect the growth of some NFPAs, there remains a high unmet need for more effective medical treatments (1, 19, 21).

Investigation of chimeric compounds that combine both a DA moiety and a peptidic SST moiety within the same compound, and that retain the ability to potently bind both to DR and SSTR, has shown promise in suppressing secretion of growth hormone (GH) and prolactin (PRL) from functioning pituitary tumors (22, 23). The enhanced efficacy of these compounds may be related to homodimer and heterodimer receptor formation between the DR and SSTRs that results in enhanced signaling. Heterodimers with enhanced functional activity have previously been observed to form between the DA and SST receptors (24, 25). Specifically, ligand-dependent heterodimerization was shown to occur between human D2R and SSTR2 in multiple cell lines (24). Similar dimeric receptor interactions have also been observed with several other classes of G-protein–coupled receptors, including the opiates and somatostatin receptors (26-28). TBR-760 (previously BIM-23A760), a chimeric DA-SST analogue compound with high affinity both for D2R and SSTR2, has demonstrated significant suppression both of GH and PRL secretion from cultured primary human acromegalic tumor cells, with both potency and efficacy significantly greater than pure DA and SST analogues, either alone or in combination (23, 29, 30). Additionally, TBR-760 was shown to activate antiproliferative pathways and to inhibit the proliferation of primary cultures of human NFPA cells in vitro (15, 31).

To examine the effect of TBR-760 on NFPA growth in vivo, we used a novel animal model of NFPA, a pro-opiomelanocortin (POMC) knockout (KO) mouse in which both homozygous double KO and heterozygous KO mice develop highly aggressive NFPAs (32, 33). The NFPAs in these mice are macroscopically visible beginning at age 7 months and grow rapidly, with the majority reaching approximately 10- to 15-fold greater size by weight than the normal pituitary gland (33). As a consequence of the aggressive NFPA growth, the POMC heterozygous mice survival rate is 50% that of wild-type mice at age 42 to 45 weeks, with 100% mortality by 60 weeks (33). In the present study, we assess the ability of TBR-760 to affect the growth of the highly aggressive NFPAs that form in the POMC KO mouse model.

## Materials and Methods

### Receptor characterization

To determine whether the POMC KO mouse tumors had a similar receptor expression profile as human NFPAs and expressed the appropriate receptors to respond to the chimeric DA-SST compounds, the expression level of messenger RNA of D2R, SSTR2, SSTR3, and SSTR5 was examined. SSTR1 and 4 were not included because of the low binding affinity of TBR-760 (843 nM and > 1000 nM, respectively) (23). Additionally, BIM-23023 has a binding affinity greater than 1000 nM for SSTR1 and SSTR4 (23). Reverse transcriptase-polymerase chain reaction (RT-PCR) for each receptor was performed in triplicate using total RNA. Each sample was reverse transcribed by Moloney-murine leukemia virus RT. For negative controls, sterile water was added in place of RT. Oligonucleotide primers for the PCR amplification of D2R, SSTR2, SSTR3, SSTR5, and GAPDH (glyceraldehyde-3-phosphate dehydrogenase) were designed using the published sequences for the respective complementary DNA. Amplification was performed using *Taq* DNA Polymerase for 35 cycles.

#### Microarray samples and experimental design.

To determine how closely the spontaneous POMC KO mouse tumors resembled human NFPAs, and thereby are a representative model for studying human NFPAs, the whole genome transcriptomic profile of the POMC KO mouse tumors was compared with that of human NFPAs. To study the genomic expression of the POMC KO mouse tumors, 10 Affymetrix Mouse Genome 430 2.0 arrays (Thermo Fisher Scientific) were created from spontaneously generated pituitary tumor tissue from both heterozygote and homozygote POMC KO mice (33). Data are available through the Gene Expression Omnibus database (GSE151857).

To study the genomic expression of human NFPAs, 40 GeneChip Human Genome U133_plus 2.0 arrays (Thermo Fisher Scientific) were created from tumor tissues obtained from Massachusetts General Hospital. Use of human samples was approved by the Massachusetts General Hospital subcommittee for human studies and written, informed consent was obtained from patients before samples were included in tissue bank. For both the mouse and human tumors, total RNA was extracted using TRIzol (Invitrogen, Thermo Fisher Scientific) according to the manufacturer’s protocol. RNA concentration was measured by ND-1000 Spectrophotometer (NanoDrop, Thermo Fisher Scientific) and RNA quality was assessed with a 2100 Bioanalyzer (Agilent), and RNA integrity number values were determined. RT of equal amounts of RNA from each sample was performed, followed by hybridization to the Affymetrix GeneChip (Thermo Fisher Scientific). Transcriptomic profiling was performed on the Affymetrix platform (Thermo Fisher Scientific) according to the Affymetrix protocol on samples with RNA integrity number greater than 7. Posthybridization, washing, and staining of arrays was performed in an Affymetrix GeneChip Fluidics FS450 station followed by scanning using an Affymetrix GeneChip 3000 7G Scanner and Affymetrix Command Console (Thermo Fisher Scientific).

##### Microarray normalization, quality assessment, and analysis.

Technical quality of the arrays was assessed using GeneSpring GX software (version 9.2; Agilent Technologies) to detect low-quality arrays by analyzing the percentage of probes detected on each array and the 5:3 signal ratio of specific marker probes. The arrays were then normalized using the Robust Multichip Average algorithm built in the GeneSpring GX software. The MAS5 absolute call filter implemented in the Affymetrix GeneChip Operating Software (was used to determine present calls of the probes in the arrays. All probes showing absent calls were removed from further analysis. Principal component analysis was then performed across all genes and samples to identify potential outlier samples.

##### Conversion of probe sets for comparison.

**Figure 1.**
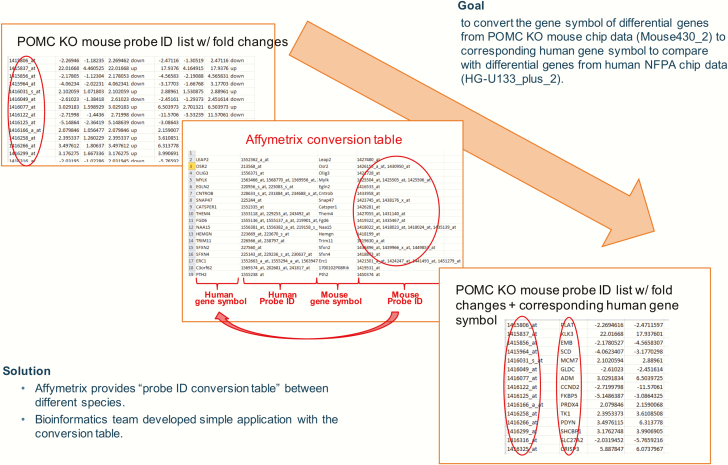
Comparison of human nonfunctioning pituitary adenomas (NFPAs) vs pro-opiomelanocortin (POMC) knockout (KO) mouse tumors: workflow.

To compare the differential genes from the 2 different species, probe set conversion was performed. The gene symbols of differential genes from the POMC mouse chip data (Mouse430_2) were converted to corresponding human gene symbols of the MGH human NFPA chip data (HG-U133_plus_2) using the Affymetrix probe identification conversion table between different species together with an internally developed application (Fig. 1).

#### Differential expression analysis.

In the POMC analysis, the differential genes were generated by comparing to pooled normal mouse pituitary. In the human NFPA analysis, the differential genes were generated by comparing to pooled human normal pituitary (Clontech Laboratories, now Takara Bio USA) and followed by subtracting marker genes such as *PRL*, *GH1*, and *POMC*from other pituitary subtypes. GeneSpring GX software was used to identify differential genes using a one-way analysis of variance (ANOVA) model with a false discovery rate of *P* less than .05 for multiple testing. Genes expressed significantly differentially between pituitary adenoma and normal pituitary tissues were generated with a fold-change cutoff of greater than 2.0 for further analysis. Pathway enrichment of the differential genes identified from POMC KO tumors and NFPAs was carried out using MetaCore (Clarivate Analytics). To evaluate the similarity of the expression pattern of the common genes, hierarchical clustering was performed on the 154 genes using GeneSpring GX software.

### Animals

All animal experiments were approved by the Duke University Institutional Animal Care and Use Committee and were in compliance with the US National Research Council’s Guide for the Care and Use of Laboratory Animals and the US Public Health Service’s Policy on Humane Care and Use of Laboratory Animals. Breeding pairs of heterozygous POMC mutant mice between ages 2 and 6 months were used to generate male and female heterozygous and wild-type animals (34). Genotyping was carried out by PCR as described (33). Mice were housed in same-sex groups of 3 or 4 animals per cage throughout the study.

### Compounds

The DA-SST chimeric compound, TBR-760 (formerly BIM-23A760), the individual SST analogue, BIM-23023, and the DA analogue, BIM-53097, were used. The binding and agonist activity at human D2R and SSTR2 for all of the compounds are shown in Table 1. The compounds were dissolved in 2% Solutol HS15 (w/w in water; Millipore Sigma 42966, Merck) and filtered using a 0.22-µm polyvinylidene fluoride filter (EMD Millipore, Cole-Palmer). Adjusting for peptide content, solutions were prepared to administer the desired doses at 0.15 mL per 30-g mouse. Solutions to deliver final concentrations for study 1 (all TBR-760) of 12.5 mg/kg, 1.25 mg/kg, and 0.125 mg/kg, and for study 2 of 1 mg/kg (TBR-760), and TBR-760 molar equivalent doses of 1 and 10 mg/kg of BIM-23023, 2 and 20 mg/kg BIM-53097 (reflecting the fact that TBR-760 has 2 DA moieties for each SST moiety), and a combination of 1 mg/kg BIM-23023 + 2 mg/kg BIM-53097, were aliquoted into Eppendorf tubes and stored at –80°C. For dosing, vials were brought to room temperature, solutions were mixed thoroughly, and injected subcutaneously using a 0.5-mL insulin syringe.

**Table 1.** Receptor binding affinity and agonist activity at human dopamine type 2 and somatostatin type 2

Compound	D2R binding, Ki nM	D2R agonist activity cAMP, EC_50_ nM	SSTR2 binding, Ki nM	SSTR2 agonist activity cAMP, EC_50_ nM
TBR-760	12.1^*a*^	0.064^*a*^	0.01^*a*^	1.22^*a*^
BIM-23023	–	–	0.42^*b*^	0.14^*b*^
Octreotide	–	–	0.43^*b*^	0.2^*c*^
BIM-53097	22.1^*b*^	0.03^*b*^	–	–
Cabergoline	0.8^*a*^	0.026^*a*^	–	–

Abbreviations: cAMP, 3′,5′-cyclic adenosine 5′-monophosphate; D2R, dopamine type 2 receptor; SSTR2, somatostatin type 2 receptor.

^*a*^Internal Tiburio data.

^*b*^(35), 2018.

^*c*^Ipsen historical data.

### Mouse studies design

Wild-type mice (3 male, 3 female) at age 6 months were scanned by MRI to determine average normal pituitary size. Heterozygous POMC KO mice were screened using MRI starting at average age 6.5 months (range, 5.5-9.5 months). These screening scans served to select animals for the study once reaching the baseline tumor volume of 10 mm^3^ and to ensure reasonably homogenous groups. Most animals developed tumors within an average of 1.5 months after their first scan and at that point were randomly assigned to experimental groups. Two studies were conducted, the first comparing several doses of TBR-760 vs vehicle, and a second study comparing a fully effective dose of TBR-760 vs the molar equivalent and the 10× molar equivalent (based on the dose of TBR-760) of a pure SST agonist (BIM-23023), a pure DA agonist (BIM-53097), and the combination of the two. The concentration of the DA agonist was doubled to reflect the 2:1 ratio of DA:SST moieties present in TBR-760.

All groups were composed of 3 males and 3 females, with the exception of the vehicle- and TBR-760-treated groups (n = 8, 4 males/4 females) of the second study, which were maintained for survival analysis as described later. Once entered into the study, all mice received MRI scans for baseline, followed by scans at 2 weeks, 1 month, and 2 months after the baseline scan. Once tumor volume reached 10 mm^3^ in the heterozygous POMC KO mice, the mice were treated by daily subcutaneous injection, with each injection given at the same approximate time of day. The mice were euthanized 8 weeks after start of treatment (ie, after the last scan), and pituitary tissue was collected, weighed, and flash-frozen, with the exception of the TBR-760 and vehicle-treated groups in the second study, which were maintained for survival analysis.

### Magnetic resonance imaging screening

All studies were conducted on a Bruker 7T/210 mm Biospec system (Bruker) at the Duke Center for In Vivo Microscopy. The initial screening scans, conducted at age 6 months in wild-type mice, and average age 6.5 months (range, 5.5-9.5 months) in heterozygous POMC KO mice, were fast scans with scan times of 4 minutes (rapid acquisition with refocused echoes [RARE], repetition time [TR] = 1 second, echo time [TE] = 50 ms; 100 μm × 100 μm × 0.4 mm). Images were analyzed in ImageJ (36). Treatment monitoring MRI scans were carried out at 2 weeks, 1 month, and 2 months after start of treatment. For all monitoring scans, parameters were 40-minute scan time (RARE; TR = 5 seconds, TE = 50 ms; 100 μm × 100 μm × 0.3 mm). All scans included complete sectional scans of the pituitary, which were used to calculate tumor volume. MR images were preprocessed using bias field correction to account for the sensitivity profile of the receive coil, thus enhancing the tumor contrast. A trained analyst then manually segmented the tumor on each slice of the MRI. Volume of the tumor was then calculated by multiplying the area of the segmentation by the slice thickness for each slice of the image.

### Survival analysis

In the second study, the survival of TBR-760–treated mice was examined and compared with vehicle-treated mice. After the initial 8-week treatment period was complete, vehicle-treated mice were paired with TBR-760–treated mice, and treatment continued as before. When a vehicle-treated mouse died, its TBR-760-treated mouse partner continued to receive treatment for an additional 8 weeks before being euthanized. On death or euthanasia, pituitaries were collected, weighed, and flash-frozen.

### Statistical analysis

Statistical analysis was performed using PRISM8 (GraphPad 2018). An ANOVA was performed followed by post hoc analysis with Dunnett’s multiple comparisons test. Results are expressed as mean ± SEM. *P* less than .05 was considered statistically significant. Survival analysis was performed using PRISM8 using the Gehan-Breslow-Wilcoxon test to compare the survival curves.

### Pharmacokinetic analysis

To study and compare the pharmacokinetic behavior of TBR-760, the DA analogue BIM-53097 and the SST analogue BIM-23023 solutions with appropriate concentrations were prepared as described earlier and mice were injected subcutaneously at time 0 with a dose of 4 mg/kg. Blood samples were collected at 15 and 30 minutes, and 1, 2, 4, 6, 8 and 24 hours after injection, and processed to obtain plasma. The determination of TBR-760, BIM-23023, or BIM-53097 was based on 100 μL plasma volume and was carried out by high-pressure liquid chromatography tandem mass spectrometry (HPLC-MS/MS) after protein precipitation with acetonitrile and subsequent solid-phase extraction. HPLC-MS/MS was performed on an AB SCIEX API 4000 LC-MS/MS with electrospray ionization operated in multiple-reaction monitoring mode for the analysis (Waters Corporation). Pharmacokinetic parameters were calculated by noncompartmental analyses using WinNonLin (version 5.2).

## Results

### Receptor and genomic expression analysis of pro-opiomelanocortin knockout mouse adenomas

**Figure 2.**
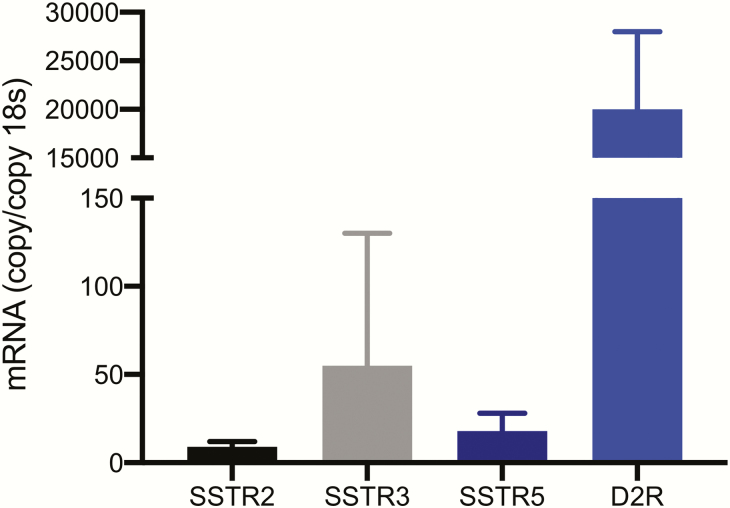
SSTR2 and D2R receptor expression in POMC knockout mouse tumors. Data are presented as mean ± SD, n = 11 tumors. SSTR, somatostatin receptor; D2R, dopamine receptor; POMC, pro-opiomelanocortin.

**Figure 3.**
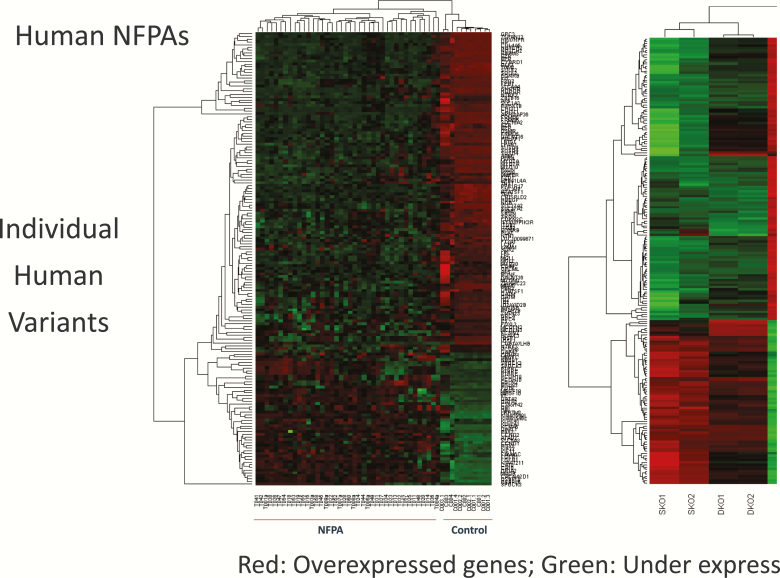
Hierarchical clustering: striking similarity in expression pattern of 154 common genes between human nonfunctioning pituitary adenoma (NFPA) and pro-opiomelanocortin (POMC) knockout (KO) mouse tumors.

To determine the relevance of POMC KO mice as a model for studying the effect of the chimeric compound TBR-760, as well as DA and SST analogues on human NFPAs, the expression of D2R, SSTR2, SSTR3, and SSTR5 messenger RNA was examined. The tumors displayed high expression of D2R (20 000 ± 8000 copy/copy 18s) and moderate expression of SSTR3 (55 ± 75 copy/copy 18s), with lower levels of SSTR2 and SSTR5 (9 ± 3 and 18 ± 10 copy/copy 18s, respectively; Fig. 2). This is similar to the expression profile observed in human NFPAs (15, 18). Microarray analysis both of human NFPAs and POMC KO tumors was performed and compared (Fig. 3). Hierarchical clustering showed a strikingly similar expression pattern in 154 common genes between human NFPA and POMC KO mouse tumors (Fig. 3).

### Microarray gene expression comparison between pro-opiomelanocortin knockout model and human nonfunctioning pituitary adenomas

**Figure 4.**
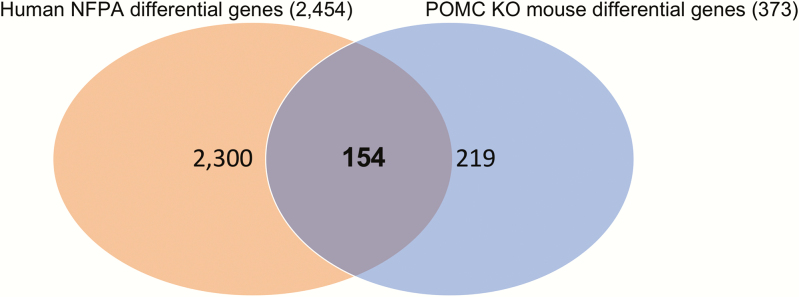
Comparison of human nonfunctioning pituitary adenoma (NFPA) differential genes with pro-opiomelanocortin (POMC) knockout (KO) mouse differential genes. Human NFPA differential genes: 3272 probes (2454 genes) generated by comparing 40 NFPA samples to pooled normal pituitary (11 technical replicates). POMC KO mouse differential genes: 425 probes (373 genes).

In the analysis of POMC KO mouse tumors, 373 genes were differentially expressed in the tumors from both homozygote and heterozygote POMC KO mice when compared to normal mouse pituitary, with a 2–fold-change cutoff (Fig. 4). The human NFPA analysis revealed 2454 genes expressed more than 2.0-fold differentially compared with pooled human normal pituitary. These 2 gene lists were compared, and 154 genes that were commonly differentially expressed both in human NFPAs and POMC KO mice tumors were identified (Fig. 4).

**Figure 5.**
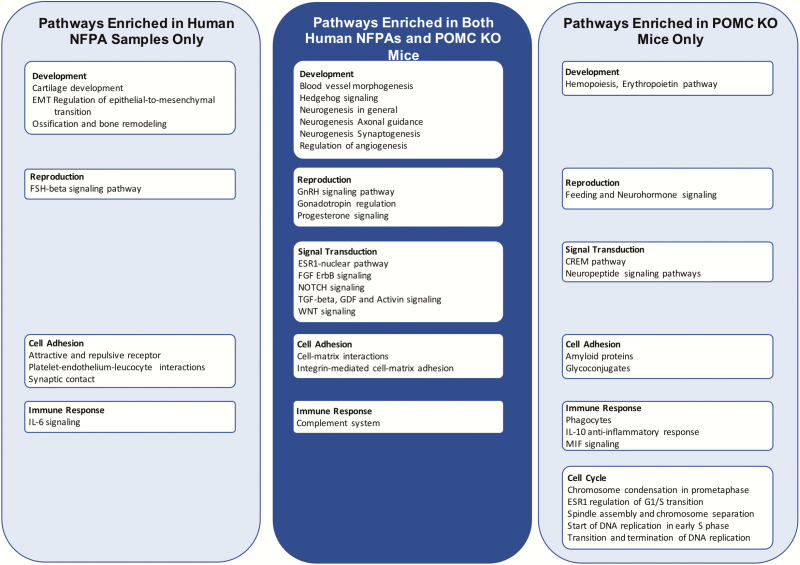
Pathways enriched in human nonfunctioning pituitary adenoma (NFPA) and pro-opiomelanocortin (POMC) knockout (KO) samples.

The 154 common genes were further analyzed for enriched functional and biological context using the MetaCore software to identify biological pathways highly associated with pituitary adenomas. Pathway enrichment analysis demonstrated that 15 pathways in 5 distinct categories were significantly enriched both in the human NFPAs and in the POMC KO tumors (*P* < .001) (Fig. 5).

**Figure 6.**
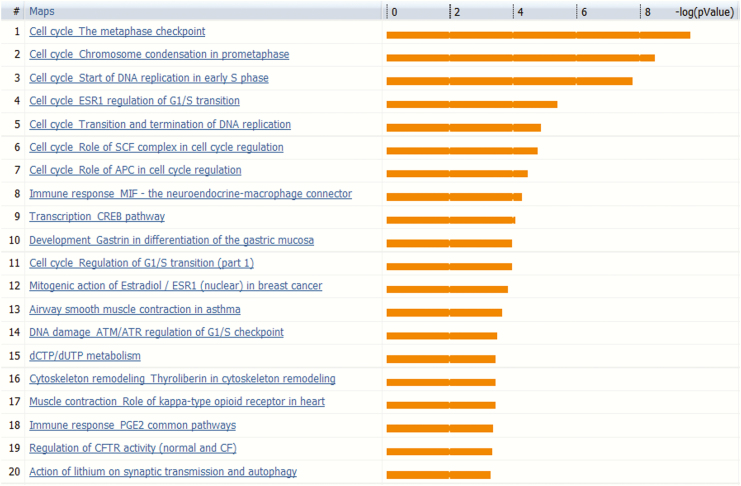
Canonical pathways enriched in 219 genes specific for pro-opiomelanocortin (POMC) knockout (KO) tumors: potential aggressive tumor pathways. The gene enrichment analysis was performed to identify canonical pathways highly associated with 219 differential genes. More than half of the top 20 pathways were related to cell proliferation: possible relevance to aggression.

Gene enrichment analysis of the 219 genes differentially expressed identified several canonical pathways, most of which were associated with cell cycle regulation and were unique to the POMC KO tumors (Fig. 6).

### Study 1: effect of TBR-760 on tumor growth in pro-opiomelanocortin knockout mice in vivo

**Figure 7.**
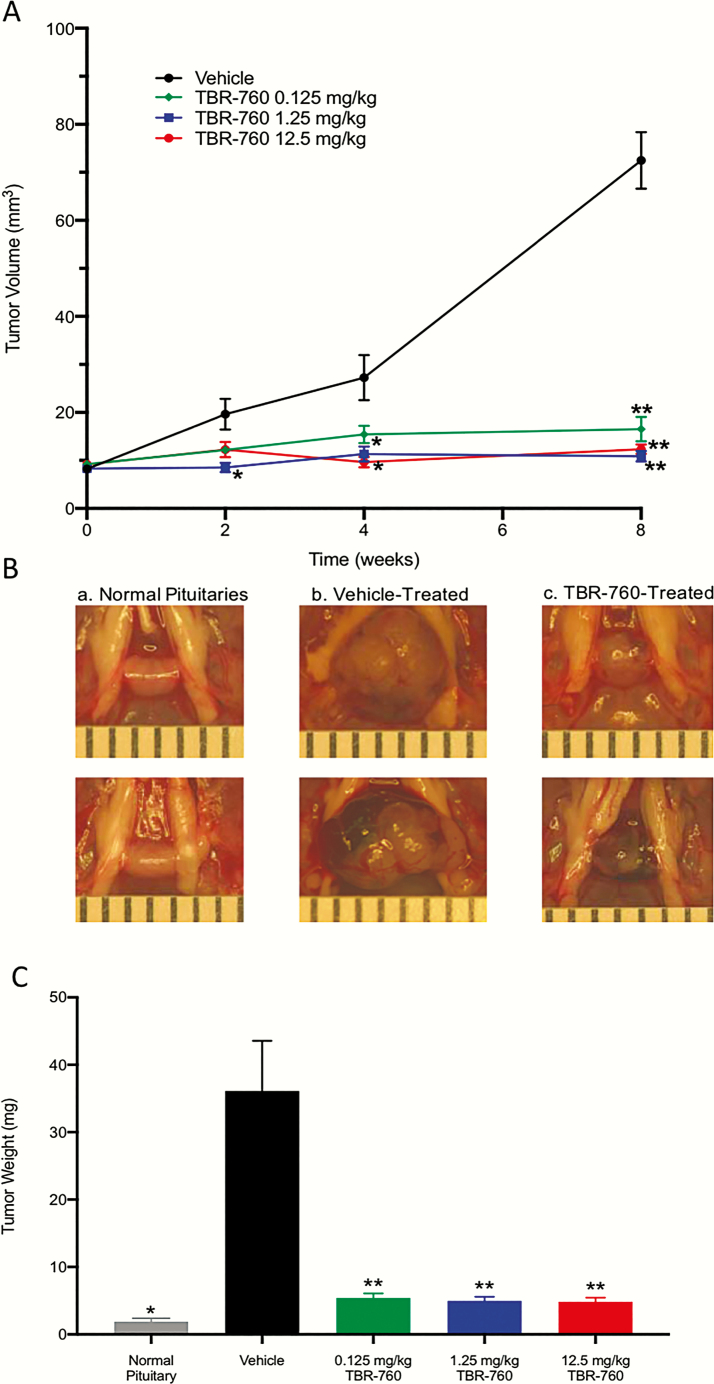
TBR-760 suppresses nonfunctioning pituitary adenoma growth in vivo. A, Tumor volume (mm^3^) over the 8-week treatment period. Data presented as mean ± SEM. *Significantly different from vehicle, *P* less than .05, n = 6. **Significantly different from vehicle *P* less than .003, n = 6. B, Photos of age-matched mouse pituitaries. a, Normal pituitaries, b, vehicle-treated, and c, TBR-760 treated (12.5 mg/kg) at the end of the 8-week treatment period. C, Tumor weight (mg) after 8 weeks of treatment (n = 6, except normal pituitary, n = 2). Data presented as mean ± SEM. *Significantly different from vehicle, *P* less than .02. **Significantly different from vehicle, *P* less than .001.

To establish whether TBR-760 had an effect on tumor growth in heterozygous POMC KO mice and to determine appropriate doses, mice (n = 6: 3 male and 3 female) were randomly assigned to treatment groups when their pituitary tumor volume reached approximately 10 mm^3^ as measured by MRI. At that time, treatment was initiated with daily subcutaneous injections of either vehicle or 0.125, 1.25, or 12.5 mg/kg of TBR-760 for 8 weeks. Mice treated with vehicle demonstrated dramatic tumor growth during the 8-week treatment period, increasing in volume from a mean of 8.2 ± 0.8 at baseline to 72.5 ± 5.9 mm^3^ (an increase of 890 ± 0.7%) (Fig. 7A). In contrast, treatment for 8 weeks with all doses of TBR-760 resulted in tumor volumes that were not significantly different from the baseline volume but were significantly different from the tumor volumes in vehicle-treated mice: 16.5 ± 2.6 mm^3^ (0.125 mg/kg TBR-760), 10.9 ± 1.1 mm^3^ (1.25 mg/kg), and 12.3 ± 1.0 mm^3^ (12.5 mg/kg) vs 72.5 ± 5.9 mm^3^ in vehicle-treated mice (Fig. 7A). The dramatic difference in tumor growth between vehicle- and TBR-760–treated mice was further confirmed by visual comparison and measurement of weights of pituitaries on death of the mice (Fig. 7B and 7C). The mean pituitary weight of age-matched wild-type mice was 1.9 ± 0.5 mg, which was significantly smaller than the tumor weight of heterozygous mice treated with vehicle (36 ± 7.4 mg, *P* < .007). In all the TBR-760-treated groups, the tumor weight was significantly different from the vehicle-treated mice, 5.4 ± 0.67 mg (0.125 mg/kg), 5.0 ± 0.61 mg (1.25 mg/kg), 4.8 ± 0.61 mg (12.5 mg/kg) vs 36 ± 7.4 mg in vehicle-treated mice (*P* < .001) (Fig. 7C).

### Study 2: comparison of individual somatostatin and dopamine agonists vs TBR-760 in arresting tumor growth

**Figure 8.**
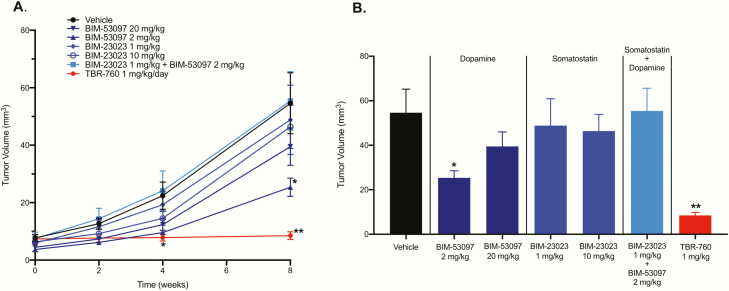
TBR-760 arrests tumor growth compared to its dopamine (DA) and somatostatin (SST) components administered both along with and in combination. A, Tumor volume (mm^3^) from treatment initiation through end of treatment after 8 weeks. B, Tumor volume at the 8-week time point. Data presented as mean ± SEM. DA, BIM-53097; SSTA, BIM-23023. *Significantly different from vehicle, *P* less than .05, n = 6-10. **Significantly different from vehicle *P* less than .001, n = 10.

**Figure 9.**
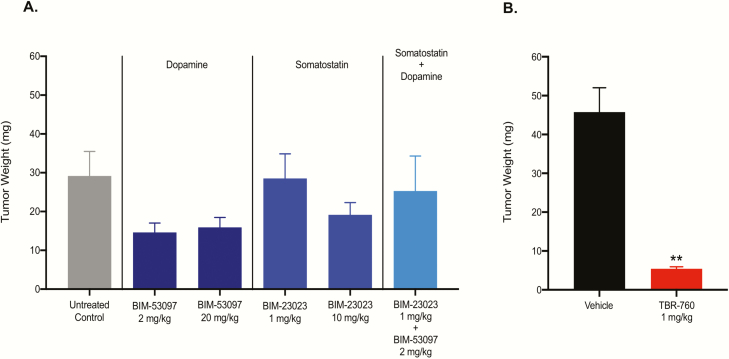
Tumors from mice treated with TBR-760 were significantly smaller than tumors from vehicle-treated mice. Treatment with either dopamine or somatostatin agonists given alone or in combination had no significant effect on tumor size. A, Tumor weight (mg) of tissue that was collected at the end of the 8-week treatment period and compared with tumors from untreated control (pro-opiomelanocortin knockout) mice. B, Tumor weight of tissue that was collected from the vehicle-treated group at the time of death and for the TBR-760–treated group 2 months after the death of its vehicle-treated littermates. Vehicle mean treatment duration was 98 ± 14.7 days and TBR-760 mean treatment duration was 156 ± 13.7 days. Data presented as mean ± SEM. **Significantly different from vehicle *P* < 0.001, n = 10.

To compare the ability of TBR-760 to arrest aggressive pituitary tumor growth in the POMC KO mouse model vs its individual SST or DA components, either alone or in combination, heterozygous POMC KO mice were again randomly assigned to treatment groups when they presented with tumors approximately 10 mm^3^ in size as determined by MRI. The individual DA analogue, BIM-53097, and the individual SST analogue, BIM-23023, were administered at equimolar and 10× doses of TBR-760 (2 or 20 mg/kg DA analogue and 1 or 10 mg SST analogue, reflecting the 2:1 DA:SST moieties of TBR-760). As observed in the earlier study, TBR-760 at a dose of 1 mg/kg/day completely arrested tumor growth so that it was maintained at the baseline tumor volume after 8 weeks of treatment with TBR-760, 8.5 ± 1.3 mm^3^ vs 54.6 ± 10.6 mm^3^ in vehicle-treated mice (*P* < .001; Fig. 8). In contrast, mice treated with vehicle once again displayed highly significant tumor growth during the 8-week treatment period (from 7.7 ± 1.1 at baseline to 54.6 ± 10.6 after 8 weeks: 612.1% increase). In 20% of TBR-760–treated mice, the tumors shrank by a mean of 44.7%. Mice treated with either dose of the SST analogue, the higher 10× molar dose of the DA analogue, and the combination of the 2, all failed to show significant effects on tumor growth, with tumor volumes after 8 weeks similar to that observed in the vehicle-treated animals (Fig. 8B). The one exception was the mice treated with the low (equimolar) dose of the DA analogue, which showed a decrease in tumor growth that was significantly different from vehicle (25.4 ± 3.2 mm^3^); however, the effect was much less dramatic than the complete arrest observed with TBR-760 treatment (Fig. 8). The pituitary weights of all animals except TBR-760– and vehicle-treated were taken after the 8-week study and compared with the pituitary weight of untreated, tumor-bearing controls. The pituitary weights in DA agonist-treated, SST agonist-treated, and the combination were not significantly different from the pituitaries from untreated, tumor-bearing controls after 8 weeks (Fig. 9A). The small yet significant decrease in tumor volume seen in the low-dose DA agonist-treated mice was not reflected in the final pituitary weight (Fig. 9A), suggesting that the volume measurements in this particular group may have been anomalous, whereas the inhibitory effect of all the other treatment groups on pituitary weights is consistent with the effect on tumor volumes.

### Survival of TBR-760–treated vs vehicle-treated mice

**Figure 10.**
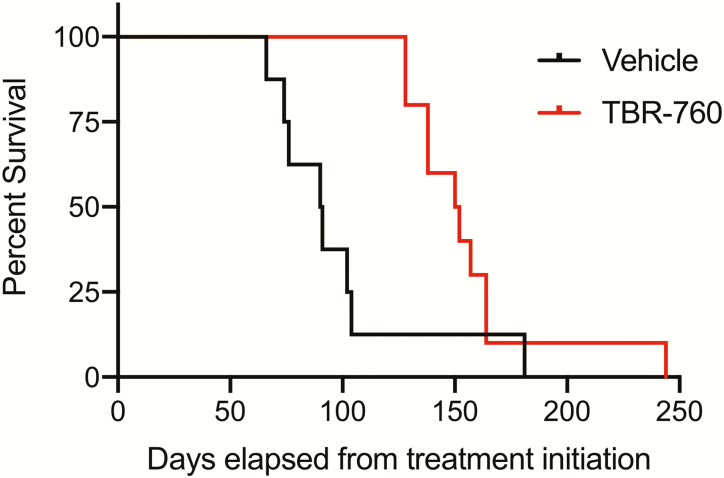
TBR-760 treatment prevents death in tumor-bearing mice. Survival curves comparing vehicle- and TBR-760-treated mice. TBR-760–treated mice were euthanized approximately 60 days after the spontaneous death of a vehicle treated littermate, n = 10. Curves are significantly different, *P* less than .02, n = 10.

Mice treated with vehicle died at an average age of 12 months (range, 11-14.5 months), whereas all TBR-760–treated mice survived an additional 8 weeks (at which time they were euthanized) past the spontaneous death of their vehicle-treated paired mouse (Fig. 10). The TBR-760–treated mice survived significantly longer than the vehicle-treated mice (*P* < .002), with a mean survival duration for the vehicle-treated mice of 98 ± 12.7 days and 156.3 ± 10.6 days for the TBR-760–treated mice. At the time of spontaneous death in the vehicle-treated group, and the time of death in the TBR-760–treated group, tumor volumes were 54.61 ± 10.61 vs 8.50 ± 1.34 mm^3^, respectively. (Fig. 8B). The final tumor weights of TBR-760–treated mice and vehicle-treated mice were significantly different: 5.42 ± 0.52 vs 45.76 ± 6.28 mg, respectively (*P* < .001)(Fig. 9B).

### Pharmacokinetics of TBR-760, the dopamine analogue, and the somatostatin analogue

**Figure 11.**
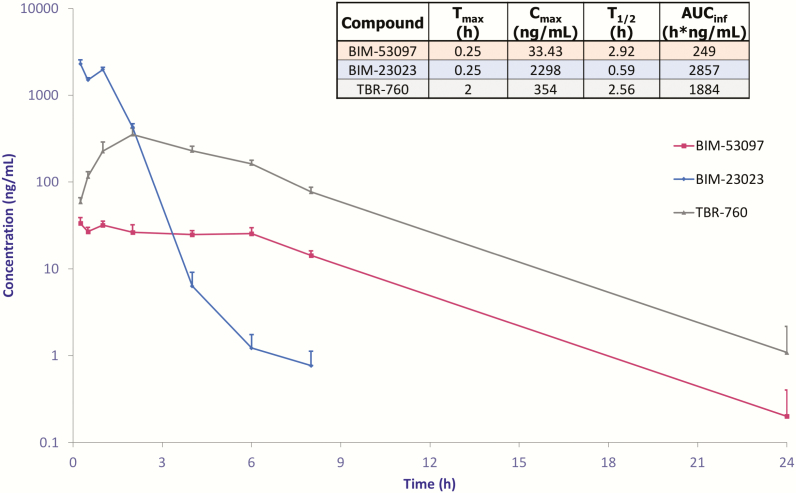
Subcutaneous pharmacokinetic profile. Pharmacokinetics curves after subcutaneous administration of TBR-760, dopamine agonist (BIM-53097), and somatostatin agonist (BIM-23023) in pro-opiomelanocortin knockout mice. Data are expressed as plasma concentration (ng/mL), n = 4.

Pharmacokinetic characteristics of TBR-760, its component DA analogue, and SST analogue, were analyzed after a 4-mg/kg subcutaneous dose. The SST analogue displayed fast absorption, resulting in a high C_max_ and comparatively faster clearance than the DA analogue or TBR-760 (Fig. 11). In contrast, the DA analogue displayed a slow and sustained absorption resulting in a much slower clearance. These characteristics appear to merge in the TBR-760 pharmacokinetic profile, with faster absorption and higher C_max_ than the DA analogue, but much slower, prolonged absorption and clearance as compared with the SST analogue.

## Discussion

The lack of medical therapy for patients with NFPAs exposes them to compounding comorbidities from the current reliance on neurosurgery and radiotherapy both as first-line and subsequent therapies (7, 37). These patients require lifelong monitoring for tumor recurrence or tumor remnant growth, and because first-line neurosurgical treatment is not curative in many cases, a significant number of patients undergo further neurosurgery and/or radiotherapy associated with a considerably higher degree of morbidity and mortality (38-41). Treatment with DA and SST analogues have shown some degree of efficacy in slowing progression but have not been rigorously tested in randomized clinical trials and are not approved for treatment of patients with NFPAs (1, 15, 16, 21, 42, 43).

In this study, we used a mouse model that spontaneously develops aggressive NFPAs to examine the impact of a potential new treatment modality, a chimeric DA-SST compound, on NFPA growth. The relevance of the tumors produced in this model to human NFPAs was demonstrated by similar differential expression of 154 genes belonging to common pathways. These gene expression similarities are intriguing given that the POMC KO mouse pituitary tumors appear to develop from melanotrophs, whereas human NFPAs originate from gonadotrophs. Most interesting, although the POMC KO tumors originate from a pituitary cell lineage different from human NFPA, the POMC KO mouse tumors display enhancement of genomic pathways that are typically associated with the gonadotroph lineage of human NFPAs, including gonadotropin-releasing hormone signaling, gonadotropin regulation, and progesterone signaling.

In contrast to the typical human NFPAs, the POMC KO mouse tumors also exhibit differentially expressed genes associated with cell cycle regulation and genomic stability. Aberrant DNA replication, damage repair, and chromatin condensation are known factors that lead to genomic instability, a hallmark of tumor initiation, progression, and aggressiveness. Cell cycle genes such as cyclin A2 (*CCNA2*), G1/S-specific cyclin-E2 (*CCNE2*), and serine/threonine-protein kinase Chk1 (*CHEK1*) are aberrantly expressed. Overexpression of *CCNA2* in mammalian cells is hypothesized to lead to delayed onset of metaphase and anaphase (44). The aggressive behavior and rapid growth of the POMC KO tumors, as compared to the typically more indolent nature of human NFPAs, may be explained by the enrichment of these canonical pathways involved in cell cycle regulation and genomic stability. Additionally, genes known to be related to pituitary tumor aggressiveness, such as securin (*PTTG1*) and fibroblast growth factor receptor 4 (*FGFR4*), are also differentially expressed in the POMC KO mouse tumors (45, 46). Further, studies have shown a certain percentage (estimates range from 15% to as high as 25%-55%) of adenomas as being classified as atypical, invasive, or aggressive (47, 48). The aggressive behavior of the POMC mouse tumors may model these aggressive human NFPAs in particular.

In addition to the highly similar phenotypic profile and similarities in enhanced expression of genomic pathways that make heterozygous POMC KO mice a viable model for human NFPAs, POMC KO mouse tumors also have an SST and DA receptor expression profile similar to that of human NFPAs, with high expression of D2R and moderate expression of SSTR3 and SSTR2 (15-18). Previous studies have shown very little to no expression of DA receptors 1, 3, 4, and 5 (42). In addition, TBR-760 and its component SST agonist BIM-23023 have both been shown to have minimal binding affinity to SSTR1 and SSTR4, therefore these were not included in the study (23). The DA and SST receptor profile of the POMC KO mouse thus make it an appropriate model for testing the chimeric DA-SST compounds, and an appropriate model for ascertaining the potential effect of the chimeric compound, TBR-760, on NFPA growth.

Treatment with TBR-760 completely arrested established tumor growth in heterozygous POMC KO mice as compared with the substantial growth observed in vehicle-treated mice over the 8-week treatment period. In contrast, treatment with comparable doses of a pure DA analogue, a pure SST analogue, or the combination of the two, had no statistically relevant effect on tumor growth and were not distinguishable from vehicle-only treatment. The one exception was mice treated with the low-dose, 2 mg/kg, DA analogue, which also significantly decreased tumor growth, but much less so than the complete suppression induced by TBR-760. Interestingly, treatment with the DA analogue at the higher dose, 20 mg/kg (10× molar equivalent of TBR-760 dose), or in combination with the SST analogue, had no significant effect on tumor growth. In addition to the potent antiproliferative effect, tumor shrinkage was observed in 20% of the TBR-760–treated POMC KO mice with a mean volume reduction of 45%. Importantly, tumor shrinkage was not observed with the pure DA or SST analogue treatment, either alone or in combination.

One reason for the lack of effect of the pure SST analogue, which is hydrophilic and water soluble, is the fast absorption and consequent rapid clearance that likely prevented compound accumulation. In contrast, the pure DA analogue, which is hydrophobic, has a slow and sustained absorption and slow elimination. Consequently, the slow absorption and clearance of TBR-760 may be due to the hydrophobic nature of the DA moiety, and this may be contributing in part to its efficacy by allowing accumulation of TBR-760 over time, which allows receptor activation by both the DA moiety and the SST moiety for a prolonged period.

In vitro studies of cultured human NFPA cells treated with TBR-760 have shown a significant inhibition of cell proliferation in approximately 60% of NFPAs examined (15). Similar, although less potent, dose-dependent inhibition of cell proliferation was also observed in response to cabergoline, octreotide, and the combination of the two (15). The TBR-760 antiproliferative effect was shown to be at least partially D2R dependent (15). Peverelli et al also observed a similar inhibition of cultured human NFPA cell proliferation in response to TBR-760 and the DA agonist used in the present study, BIM-53097; however, SST2 and SST5 selective analogues had no effect (31). This antiproliferative effect, at least in part, was likely via induced apoptosis, as evidenced by an increase in caspase-3 activity, a crucial component of the apoptotic pathway (31). The mitogen-activated protein kinase pathways, extracellularly regulated kinase 1/2 and p38, were also implicated as intracellular pathways involved in the cytostatic and cytotoxic effects of TBR-760 and the DA agonist (31). Given the strong antiproliferation effects of TBR-760 observed in POMC KO mice, as well as the observed tumor shrinkage, it is plausible that these same pathways are also activated and contribute to the response to TBR-760 in the POMC KO mouse.

Beyond the rapid in vivo clearance of the pure SST analogue, the reasons why the pure DA and SST agonists inhibit NFPA cell proliferation in vitro but do not have a significant effect on tumor growth in the POMC KO mice is unknown. One possibility is the lack of competition with endogenous ligands in vitro that allows unencumbered interaction with the appropriate receptors. It is possible that in vivo competition for the monomeric receptors may limit the potency of a pure SST or DA agonist at its respective receptors, especially when rapidly cleared as in the case of the pure SST analogue. In contrast, the competition for unique binding sites on the heterodimers formed between SSTR2 and D2R may not be a factor and may allow much greater access and potency for chimeric DA-SST compounds like TBR-760. Baragli and colleagues reported that when D2R and SSTR2 form heterodimers in the presence of select ligands, increased affinity for DA and augmented signaling was observed (24). Other researchers have shown heterodimer formation between DA and SST receptors and other G-protein–coupled receptors with enhanced functional activity (25, 28). It is therefore possible that an enhanced ability of the chimeric TBR-760 to bind with greater affinity or modulate activity of these heterodimers may contribute to the greater effect on tumor growth than the DA or SST agonists alone or in combination.

The enhanced in vivo activity of TBR-760 compared to the pure DA and SST agonists may also be due to the existence of a known metabolite. In clinical trials of TBR-760 for the treatment of acromegaly, single-dose administration of TBR-760 resulted in significant, sustained suppression both of GH and PRL; however, with chronic, repeated administration the suppression of GH/insulin-like growth factor 1 gradually waned to become similar to that of SST agonists alone, whereas the suppression of PRL remained profound. It was discovered that a high concentration of a potent dopaminergic metabolite accumulated rapidly with multiple repeat doses of TBR-760 (22, 23). It is believed that the excess concentrations of the dopaminergic metabolite occupied the majority of DA receptors and prevented the formation of heterodimers between DA and SST receptors. Consequently, the ability of TBR-760 to suppress GH was impeded. Although this accumulating dopaminergic metabolite was problematic for the treatment of acromegaly, this mechanism may be aiding the in vivo suppression of NFPA growth by TBR-760. In NFPAs, expression of DA receptors is significantly higher than that of SST receptors. Consequently, it is likely that there are sufficient DA receptors to bind and respond to the high level of metabolite, while still having sufficient receptors to form heterodimers with the SST receptors and interact with the parent compound, TBR-760. The antiproliferative response of NFPAs to dopaminergic agonists has been demonstrated both in vitro (15, 31) as well as in clinical studies (19, 21). The full contribution of the metabolite to the combined effect with the parent TBR-760 compound on NFPA growth in vivo cannot be fully quantified; however, the present study clearly demonstrates that TBR-760 potently and completely arrests aggressive NFPA growth in the POMC KO mouse model.

The impact of TBR-760 suppression of NFPA growth in the POMC KO mouse was further demonstrated by the prevention of tumor-induced mortality. Mice treated with vehicle died at an average age of 12 months, whereas all TBR-760–treated mice survived as long as TBR-760 treatment was maintained, and at least until death 8 weeks after the spontaneous, tumor-induced death of their vehicle-treated paired mouse. Although it is not known how long the mice would have lived with continued TBR-760 treatment, it is unlikely that tumor growth would have been a contributing factor to their eventual death.

Possible limitations of the study include a lack of understanding as to the mechanism by which deletion of one or both copies of the *POMC* gene results in the consistent, spontaneous development of aggressive, nonfunctioning pituitary adenomas. Another possible limitation is that tumors in the POMC KO mice appear to originate from melanotrophs and not gonadotrophs, as in humans. However, the genomic and receptor expression profiles both of the POMC KO mouse and human tumors are very similar, and certain genes associated more with gonadotrophs than melanotrophs are expressed in the POMC KO mouse tumors.

This study has established the POMC KO mouse as a highly relevant model for the study of human NFPAs. It has also demonstrated that treatment with the DA-SST chimeric compound TBR-760 arrests and shrinks established NFPAs in POMC KO mice. Considering the unmet medical need due to the limited and less-than-optimal treatment options for patients with NFPAs, the results of this study support the clinical evaluation of TBR-760 in patients with NFPA. The potential to develop TBR-760 as a therapy for patients with NFPAs as an alternative to invasive repeat surgery and irradiation and to help avoid well-documented comorbidities of the disease and current treatment options is an exciting and important prospect.

## Abbreviations

D2R: dopamine type 2 receptor
DA: dopamine
GH: growth hormone
KO: knockout
NFPA: nonfunctioning pituitary adenoma
PCR: polymerase chain reaction
POMC: pro-opiomelanocortin
PRL: prolactin
RT: reverse transcriptase
SST: somatostatin
SSTR2: somatostatin type 2 receptor
TSS: transsphenoidal surgery

## Additional Information

***Disclosure Summary:*** H.A.H. is employed by Tiburio Therapeutics, U.H. has nothing to declare, R.N. has nothing to declare, A.T. and J.K.R. are employed by Ipsen Bioscience and own stock, G.A.J. is an employee of Duke University Medical Center, and M.D.C. is a consultant to Tiburio Therapeutics and chief scientific officer for Amolyt Pharma. All authors have submitted the International Committee of Medical Journal Editors form for disclosure of potential conflicts of interest. Conflicts that the editors consider relevant to the content of the manuscript have been disclosed.

***Data Availability:*** Restrictions apply to the availability of data generated or analyzed during this study to preserve patient confidentiality or because they were used under license. The corresponding author will on request detail the restrictions and any conditions under which access to some data may be provided.
